# Magnetic Domains and Their Power Spectral Densities in Non-Oriented Electrical Steel after Thermal Compression at Different Rates

**DOI:** 10.3390/ma16155311

**Published:** 2023-07-28

**Authors:** Yuqi Wang, Zhenyu Gao, Li Luo, Chunmei Chen, Zhiyang Zhao, Renbo Song, Yingchao Zhang

**Affiliations:** 1 Angang Steel Co., Ltd., Anshan 114021, China; 2 School of Materials Science and Engineering, University of Science and Technology Beijing, Beijing 100083, China

**Keywords:** magnetic domain, non-oriented electrical steel, thermal deformation, MFM, PSD

## Abstract

The magnetic domains of non-oriented electrical steel bearing cumulative thermal compressions made by a Gleeble 3500 Thermal System were observed using an atomic force microscope (AFM). The component forces, comprising the magnetic forces between the AFM probe and magnetic domains of the samples, along the freedom direction of the probe, were measured, and they formed the value fluctuation of the magnetic domains. The fluctuations of the magnetic domains were analyzed by examining the power spectral density (PSD) curves. The hysteresis curves of the samples were measured using a highly sensitive magnetic measurement system. An analysis of the magnetic force microscope (MFM) maps suggested that some magnetic domains were compressed into crushed and fragmented shapes, similar to the microstructure of deformed grains. Meanwhile, some were reconstructed within the thermal compressions, like dynamic recrystallization microstructures. Meaningfully, the MFM probe moved and deformed the proximal magnetic domains of tested samples within the region of its weak magnetic field. The peak positions of the magnetic domains with a high deformation rate were shifted and moved during the measuring processes by the weakly polarized probe. Both windward and leeward sides simultaneously expressed a slope towards each co-adjacent valley in the MFM maps and induced a statistical throbbing within a narrow band in the PSD curves. Thus, the MFM scanning mode was also analyzed and improved to obtain accurate MFM maps with low disturbances from the weak magnetic field of the probe. Swapping the order positions of the middle processes in the MFM scanning and adding a gliding step between them could offset the peak skewing of magnetic domains caused by the weakly polarized probe during MFM measurement process without incurring excessive replacement costs. Accumulative compression at a high rate (10 s^−1^) would crush magnetic domains into irregularly decreasing sizes with messy boundaries. This investigation provides an example of the complete relationships among deformations, magnetic domains, and magnetic properties.

## 1. Introduction

Both strength and iron loss are key properties of the non-oriented electrical steel used for the development of energy conversion and mechanical power source components in a wide variety of industries, including electric motors and large generators [1]. Obtaining non-oriented electrical steel with high magnetic properties is regarded as one way to reduce energy consumption by these industries [2,3]. Unfortunately, improving strength often results in increased magnetic property degradation and iron loss [4], which is known as the strength–magnetism trade-off. The magnetic property that deteriorates most distinctly is the magnetic permeability. Usually, the iron loss in non-oriented electrical steel includes eddy current loss, magnetic loss, and anomalous loss. Correspondingly, high resistivity results in low eddy current loss, while high magnetic performance results in low hysteresis loss [5,6]. Magnetic properties, including the magnetic permeability and coercive force, are affected by the magnetic domains, just as the mechanical properties are affected by the microstructure.

Currently, magnetic property measurement and texture analysis are mainly adopted in non-oriented electrical steel research. The magnetic properties of non-oriented electrical steel are still being explored [7,8] to establish a combined method for examining the magnetic domains and magnetic properties [9,10]. The magnetic domains and the magnetic properties should be observed and measured simultaneously, which is similar to the principle of obtaining the microstructure and mechanical properties together when analyzing structural steel [11,12]. In this study, PSD curves were used to quantify and evaluate MFM maps for comparison with hysteresis loops. In addition, the current observation mode of MFM scanning showed a deficiency during the observation processes on the magnetic domains. The magnetic field belonging to the probe of the MFM causes variation in the proximal magnetic domains and thus causes extra systematic errors. Therefore, improvements and guidance for avoiding model deficiency are proposed herein in order to improve measurement precision.

## 2. Experimental Procedure

The material for the investigation was non-oriented electrical steel containing C 0.0016%, Si 3.04%, Al 0.89%, Mn 0.45%, Cu 0.30%, Mo 0.11%, S 0.0023%, and P 0.0047% by weight, with Fe balance. The liquid steel was poured into a low-carbon steel cylindrical mold for natural solidification, after being stirred and melted in an electromagnetic vacuum furnace. The casting mold had a conical cavity volume of Φ180 mm × 300 mm. The ingot had a weight of approximately 48 kg after its oxide surface was removed. The samples were obtained from the upper one-third of the ingot by wire electrical discharge machining. The as-cast cylindrical samples (A, B, and C) with sizes of Φ8 mm × 12 mm were subjected axially to 60% cumulative deformation using a Gleeble 3500 Thermal System (Dynamic Systems Inc., Austin, TX, USA) at different deformation rates (A: 0.1 s^−1^; B: 1 s^−1^; C: 10 s^−1^) from 1050 °C, and the stress–strain curves of the deformation processes were traced. The compressed samples were longitudinally cut along their axes using a wire electric discharge cutting machine (WEDM) to observe the shaft sections. The observational transverse direction was set to the original compression direction (axial direction), and the observational longitudinal direction was set to the original radial direction. The samples for microstructure and magnetic domain analyses were etched statically in a solution of 5 vol% nitric acid and 95 vol% ethanol for 10 s at 25 °C after being mechanically polished. The microstructures were acquired by using a scanning electron microscope (SEM, Merlin Compact, ZEISS, Oberkochen, Germany), and the magnetic domains were observed by using a Dimension FastScan^©^ AFM (non-contact mode for AFM and retrace mode for MFM). The samples, cut to 4 mm × 4 mm × 1 mm by EDCM, were measured using a Quantum Design^©^ High-sensitivity Magnetic Measurement System (VSM mode, EM4-CSB magnet, 86-LC coil, Gap3-SSVT) to obtain the hysteresis curves. The testing magnetic fields were located at the 4 mm × 4 mm surfaces and perpendicular to the compression directions of the samples.

## 3. Results and Discussion

### 3.1. Magnetic Domains

**Figure 1:**
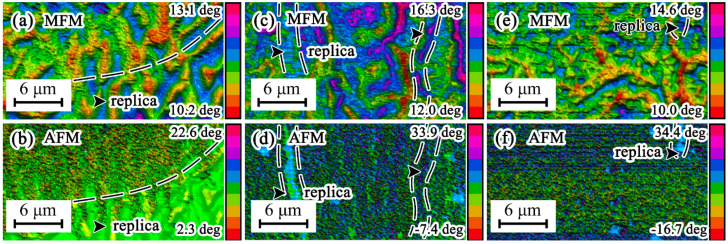
Simultaneous MFM maps and AFM microstructures under different compression rates: (**a**) 0.1 s^−1^, (**b**) 0.1 s^−1^, (**c**) 1 s^−1^, (**d**) 1 s^−1^, (**e**) 10 s^−1^, (**f**) 10 s^−1^.

Figure 1 presents the magnetic force microscope (MFM) maps and microstructures of the samples under thermal compression, obtained by the use of AFM in sequence. The magnetic domains with a medium deformation rate revealed great disparity, with both labyrinth-like and fragment-like shapes. The magnetic domains with a low deformation rate, known as ‘labyrinth magnetic domains’ [13], were observed with relatively regular shapes, large sizes, and distinct fluctuations (Figure 1a). On the contrary, the magnetic domains with a high deformation rate, known as ‘fragment magnetic domains’, were variant and strange due to the severe compression deformation (Figure 1e). It was speculated that the reference system coordinates of magnetic forces in each atom moved along with the plastic deformation, except for the continuous transition in the magnetic domain walls. The magnetic domains comprise the magnetic moments of each atom. Along with these deformations, the atoms’ locations changed in the materials. Meanwhile, the original magnetic domains moved and changed in shape because they are intrinsically related to the atoms. Acute deformations brought great change in the grains formed by these atoms. This factor acted on and intensified the transformation of the magnetic domains. Overall, the fragment magnetic domain blocks bore greater compression deformation than did the labyrinth magnetic domains; both parts were intertwined, or the fragment magnetic domains were hidden in the inner bays of the labyrinth domains.

In the AFM analysis, the atomic force and magnetic force measurements were operated successively, using a probe that had been polarized by a magnetic field, in two one-round processes. Generally, the atomic force is measured when a weakly polarized probe touches the sample surface, and the magnetic force is measured with a large distance between this probe and the sample surface [14,15]. For common samples, the AFM measurement process is performed, accompanied by MFM mapping, in tapping mode using a weakly polarized probe. This means that the motion of the weakly polarized probe with driving force should be stopped by knocking at the sample surface. However, the magnetic repulsive force between the sample surface and the probe decelerates the probe non-negligibly, causing it to stop at some distance from the sample surface when the force is too formidable. Then, the AFM records the value including the excess influence from the magnetic repulsive force as the real value, meaning that the AFM maps could be appended and overlaid by the MFM spectra. The appended region behaves like the magnetic domains with strong magnetic properties. A brightly colored region with tiny compression deformation appeared in the lower right-hand corner of the AFM map (Figure 1b) and was highly similar to the same location of the corresponding MFM map (Figure 1a). The highlighted replicated area in Figure 1a,b shows that the magnetic domains with strong magnetic force affected the AFM measurement, causing their appearance to be duplicated onto the relevant AFM map. Although this replica-area microstructure would not virtually exist on the sample surface, such a phenomenon means a magnetic domain morphology with strong magnetic properties existing in the deformed region. Contrastingly, the corresponding replicas of strip-shaped boundary microstructures in both the MFM maps (Figure 1c,e) and the AFM maps (Figure 1d,f) did not follow the principle of the replica area in Figure 1a,b. They were not formed by the magnetic force of the samples, because the replica areas in the AFM maps lacked the homologous fluctuation of the MFM maps, but instead came from the actual microstructure of the sample surfaces during AFM measurements. As the strain rate increased, the magnetic force intensity decreased along with the complex magnetic domain microstructures, weakening the impact on the AFM maps. Meanwhile, samples B and C could not disturb the AFM measurements due to their lack of magnetic force, simultaneously reflecting the strong influence of magnetic domains in sample A (Figure 1a).

### 3.2. PSD Curves

**Figure 2:**
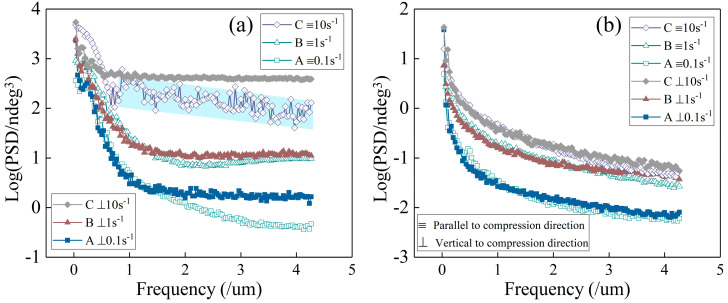
The PSD curves of (**a**) magnetic domains and (**b**) microstructure morphologies.

Each section of the stress–strain curves increased integrally as the deformation rates increased, impacting the PSD curves and the morphological parameters of the magnetic domains, besides their visual morphology. Figure 2 presents the PSD curves of the magnetic domains and microstructures observed in the thermal compression direction and the vertical direction. PSD is a probabilistic statistical method measuring the mean square value of a random variable [16,17]. In the PSD spectra, a decrease in the abscissa value signifies frequency reduction, indicating a large-span morphology hummock and a relatively flat morphology of the surveilled planes. In this study, we used graph quantification and fluctuation statistics to monitor and evaluate the magnetic domains and microstructures of the thermally treated samples.

In Figure 2b, all the microstructure PSD curves decreased rapidly from the peak value at the starting points, signifying the smoothness and quality of the sample surfaces at the micro level. The two PSD curves of each sample in the two orthogonal directions fit well, illustrating that each sample possessed similar morphological conditions in both directions. The morphology curves for the three samples formed similar trends and inflections, even under the disturbing conditions of the magnetic force carried by the weakly polarized probe. The overall level differences in the PSD curves were due to the differences among the starting point values, slightly affecting the surface morphology assessment. Contrastingly, the tendencies and difference values of each curve along the abscissa were effective for morphology analyses and were also suitable for MFM discussions. In Figure 2a, the four PSD curves detecting magnetic domains with low strain rates (0.1 s^−1^ and 1 s^−1^) possessed similar high starting points, contiguous inflections, and rapid decreasing trends. The two curves of each sample in the two orthogonal directions, consistent with the corresponding MFM maps in Figure 1, essentially kept pace with each other, showing distinct variance to those of the sample compressed rapidly (10 s^−1^).

In Figure 2a, the initial tendency of the PSD curves vertical to the compression direction with a high compression rate (10 s^−1^) declined slowly. Compared with those of other samples, this curve indicated a high proportion of magnetic domain comminutions and poor magnetic domains. Additionally, the PSD curve indicating the compression direction fluctuated in the high-frequency range of the abscissa because of the peak skewing of the magnetic domains and the direct reciprocating measurement method. The weakly polarized probe used in the MFM analysis detected a narrow strip twice in one round-trip process for the microstructural observation and then detected the same position twice again at a distance in the next round-trip process for the magnetic domain observations. The weak skewing of magnetic domains was promptly probed as a system deviation in the same direction during the second reciprocating measurement and compared to the magnetic domain measurement with assumed accuracy. In other words, the second reciprocating measurement of the magnetic domain was subject to red-shift or blue-shift phenomena, which meant that the reciprocating measurement data of the positive or negative frequency ranges presented displacements. The PSD curves perpendicular to the compression direction had no vibration, according to the indirect measurement method. Only the signal value was directly detected by the MFM probe in the reciprocating measurement parallel to the compression direction. The directly detected signals of the PSD curves perpendicular to the compression direction were superimposed and synthesized with the space coordinates. In other words, the PSD curves perpendicular to the compression direction depended on all directly detected data with common vibrations, resulting in a smooth curve.

The unidirectional unevenness curve of a strip signal is

(1) q=q(l)

where the independent variable *l* is the length, and the dependent variable *q* is the atomic force intensity, which are the length unit dimensions. The variable *n* is the spatial frequency, representing the number of atomic force wavelengths included in the unit space (1 μm). The identification frequency is 0.033 μm^−1^, guaranteeing the observation requirements for resolving the spatial frequency on the observation surfaces.

The atomic force ruggedness signal is valid only in space length, and the spatial domain finite signal is

(2) $$\begin{array}{cc} q_{L}\left(l\right)=q\left(l\right) & l\in \left[0,L\right] \end{array}$$

where *L* is the maximum space length, and *l* is the span of the fluctuation. The autocorrelation function is

(3) $$\begin{array}{cc} R_{q}\left(l\right)=\frac{1}{L}\int_{0}^{L}q\left(\tau \right)\cdot q\left(\tau +l\right)d\tau & l\in \left[-L,L\right] \end{array}$$

where *τ* is the span factor. The average power of the atomic force unevenness signal is

(4) $$\psi_{q}^{2}=\frac{1}{L}\int_{0}^{L}{\left[q_{L}\left(l\right)\right]}^{2}dl=\frac{1}{L}\left[\int_{-\infty }^{-L^{-1}}{\left|Q\left(n,L\right)\right|}^{2}dn+\int_{L^{-1}}^{+\infty }{\left|Q\left(n,L\right)\right|}^{2}dn\right]=G_{q}\left(n\right)$$

where *n* is the frequency domain and Q(n,L) is a continuous spatial domain spectrum; $n\in \left[-\infty ,-L^{-1}\right]$ represents the reverse pass measurement process, and $n\in \left[L^{-1},\infty \right]$ represents the forward pass measurement process. Further, $G_{q}\left(n\right)$ is the PSD of the spatial domain:

(5) $$G_{q}\left(n\right)=\frac{2}{L}\int_{L^{-1}}^{+\infty }\left[{\left|Q\left(n,L\right)\right|}^{2}dn\right]$$

Generally, the two equations are made equal. However, the high-frequency region of the magnetic PSD curve (with a compression rate of 10 s^−1^) fluctuated uncommonly, vertical to its compression direction. Besides this, the curve possessed a similar stabilized trend to the other curves. The genetic problem of multiple compressions was not considered for this phenomenon because it was similar and continuous in its volatility. Otherwise, the curve would develop intermittent and abrupt fluctuations. On the contrary, the possibility of the last compression causing uneven deformations and weak magnetic domains whose intensity peaks easily deviate from their center positions was considerable, as described above. The skewing of the weak magnetic domains detected by the second measurement process was caused by the high-rate compression deformations or the weak polarity of the measuring probe during the first measurement process. The red-shifted or blue-shifted frequency spectrum signal was poor in matching, causing a shift in the unidirectional frequency items on the overall observation surface of this sample (Equation (4)). It could be considered that the compression behavior of the magnetic domains exhibited a strange phenomenon at the highest strain rate (10 s^−1^) when combining the magnetic PSD curves of the three strain rates. The differences appeared not only in the size of the magnetic domains, but also between the forward and reverse measurement results of the magnetic domains in the compression direction. Uneven magnetic peak positions deviated from the center positions of the magnetic domains, affected by the exploration method, which was consistent with the observed MFM maps.

Moreover, the curves in the low-frequency range (less than 0.6 μm^−1^) were smooth, proving the robustness of magnetic domains with large spans. They were hardly affected by the weakly polarized probe; thus, the red-shift and blue-shift phenomena were inconspicuous and were diluted by the data.

### 3.3. Magnetic Hysteresis Curves

**Figure 3:**
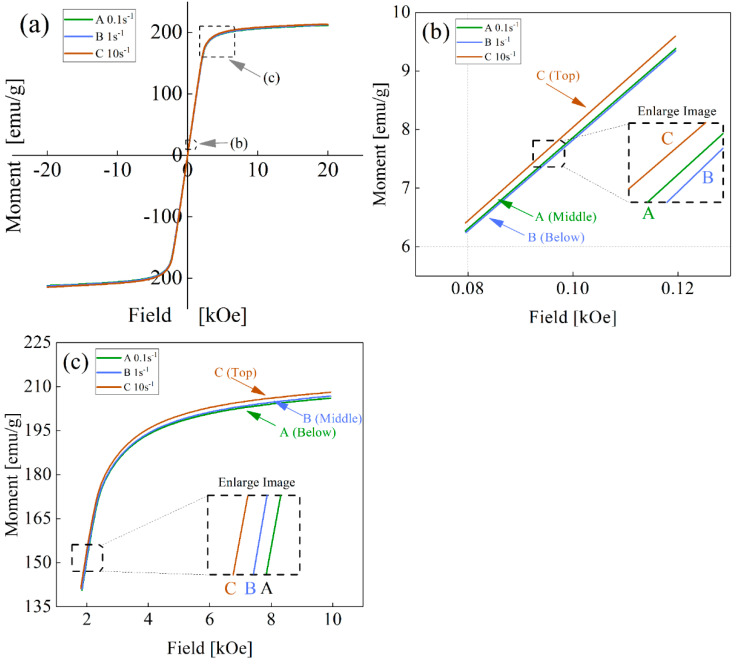
Magnetic hysteresis curves of the three samples with different deformation rates: (**a**) complete view; (**b**) low-axis region; (**c**) high-axis region.

Figure 3 presents the hysteresis curves of the three samples, showing them to be susceptible and almost inseparable, with similar weak coercive forces shown in Table 1. The differences among them could still be identified and were consistent with the magnetic domain theories of ‘labyrinth’ and ‘fragment’ above and with the PSD curve fluctuations. Sample C with a high compression rate showed greater susceptibility to magnetic fields than did the others (samples A and B) [18] due to the lower coercive forces. Magnetic materials with low coercive forces are magnetized or change their magnetized conditions under weak magnetic fields. The required magnetic field strength for the same magnetic response in sample C was lower than it was in the other samples before magnetization saturation (Figure 3b). Sample C produced a stronger magnetic response than did the others with the same magnetization saturation (Figure 3c). All demonstrated that compression deformations at a high rate prompted the magnetic domains to be easily shifted by a weak magnetic field.

### 3.4. MFM Measurement Anticipation

**Figure 4:**
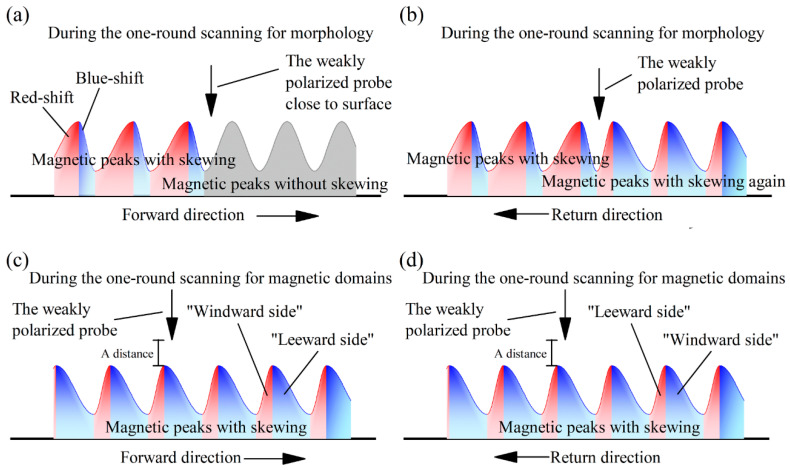
Peak skewing of magnetic domains attracted by the weakly polarized probe; the skew direction is opposite to the repulsion situation. (**a**) the magnetic behavior during the forward stage of the one-round scanning for morghology; (**b**) the magnetic behavior during the return stage of the one-round scanning for morghology; (**c**) the magnetic behavior during the forward stage of the one-round scanning for magnetic domains; (**d**) the magnetic behavior during the return stage of the one-round scanning for magnetic domains.

Figure 4 presents a longitudinal section illustration of the peak skewing process in the magnetic domains, explaining the influence of the weak polarity from the measuring probe during one measurement process of the microstructures and magnetic domains. During the MFM measurements, the component forces, comprising the magnetic forces between the AFM probe and the magnetic domains of samples, along the freedom direction of the probe, were measured, and they formed the value fluctuations of the magnetic domains. Though the magnetic domains were uniformly magnetized, the MFM could only plumb the force along the single freedom direction of its probe at one tested place, so that the magnetic domains showed high or low signal values forming peaks or valleys.

The weakly polarized probe swept from left to right (in the forward direction) and then from right to left (in the return direction) in one round of measurement of the microstructure or magnetic domains. The probe exploring the microstructure during the one-round measurement process intermittently touched the sample surface. The probe exploring for magnetic domains during the second one-round measurement process operated at a distance to the sample surface, with the height being the sum of a systematic distance and the elevation undulations of the microstructures. During the scanning processes for the microstructure, the magnetic domain peaks shifted and slid, prompted by the attraction or repulsion between the magnetic domains and the weakly polarized probe (Figure 4a,b). During the scanning processes for magnetic domains, the strength signals of magnetic domains with peak skewing were measured by the weakly polarized probe (Figure 4c,d), generating PSD fluctuations in the measuring direction (Figure 2a) as a systemic error.

In other words, the maximum intensities of magnetic domains in the tested regions usually maintained primary values, but the orientation of the magnetic forces of each atom rotated as the probe swept across. The components of the magnetic forces in the single freedom direction of the probe between the probe and magnetic domains varied, causing continuous movement of the magnetic peaks.

**Figure 5:**
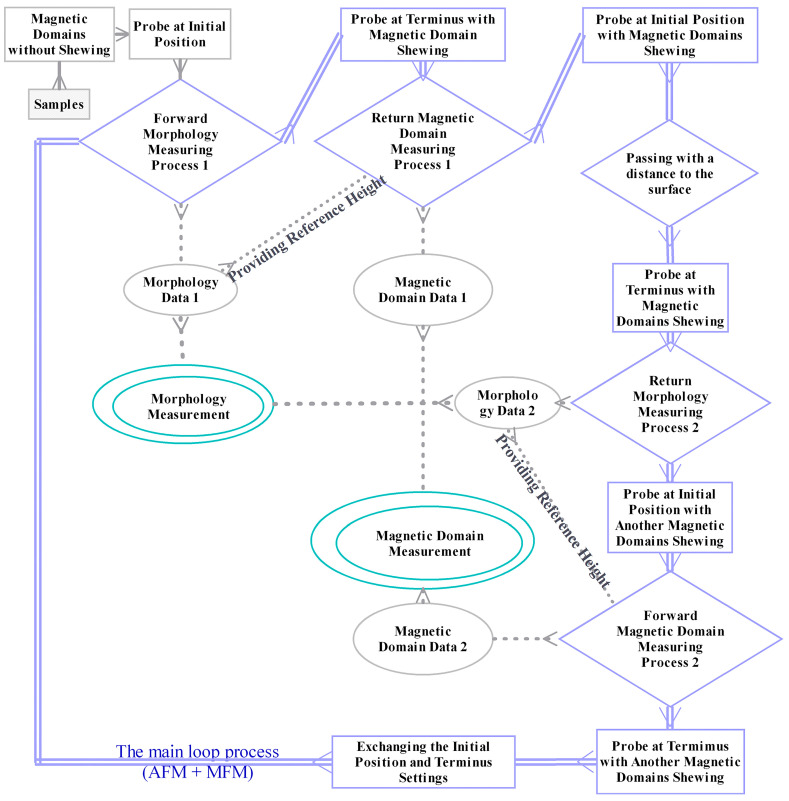
A conceptual illustration of the updated MFM measurement mode.

**Figure 6:**
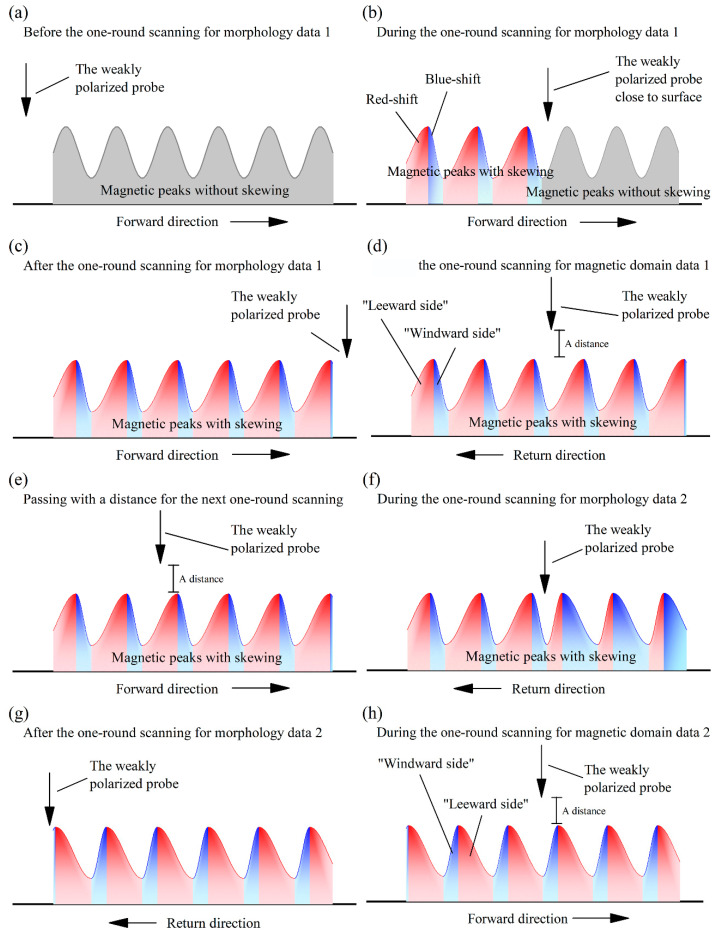
Peak skewing of magnetic domains attracted by the weakly polarized probe; the skew direction is opposite to the repulsion situation in the updated mode. (**a**) the orignal magnetic domain; the magnetic domain (**b**) during and (**c**) after the one-round scanning for morghology data 1 as long as (**d**) its during the one-round scanning for magnetic deomain data 1; (**e**) the passing process for the next one-round scanning; the magnetic domain (**f**) during and (**g**) after the one-round scanning for morghology data 2 as long as (**h**) its during the one-round scanning for magnetic deomain data 2.

Therefore, the MFM measurement mode [19] was explored and updated to correct and resolve the peak skewing of the magnetic domains—the systemic error—wherein the weak magnetic domains were shifted by the weakly polarized probe. The forward measurement processes and the backward measurement processes of the magnetic scanning are of equal cost for acquiring magnetic domains within the scanned strips. The order of the second microstructure measurement and the first magnetic domain measurement, performed during the two one-round measurement processes, could be swapped between the other morphology and magnetic domain measurements (Figure 5) to offset the magnetic domain peak skewing caused by the weakly polarized probe during the microstructure measurement processes. Then, the blue-shifted half peaks are all to the windward side and the red-shifted half peaks are all to the leeward side, synthesizing the peak position results. Figure 6 forecasts the MFM measurement process using the updated MFM measurement mode. On a certain measuring strip, the magnetic domain measurement is performed in the return process (Figure 6d) after the previous forward process for the microstructure measurement (Figure 6b), followed by one vacancy passing of the probe (Figure 6e). This passing process, performed at a distance to the measuring surface, and exchanging the initial position and terminus between the two measurement actions are necessary processes for realizing the updated measurement mode. Then, the microstructures and magnetic domains are explored sequentially in the next return and forward scanning processes (Figure 6f,h). Thus, the influence of the weakly polarized probe on the weak magnetic domains could be reduced during the total process of MFM measurement. The MFM signal data from the two directions would then be combined into the final results.

## 4. Conclusions

The magnetic domains of non-oriented electrical steels bearing compression deformations revealed synchronous decreases in size, uneven variants, and high proneness to skewing under magnetic interference:

* The thermal compression deformations affected the magnetic domains. Accumulative compression at a high rate (10 s^−1^) crushed the magnetic domains into irregularly decreasing sizes with messy boundaries.
* The weakly magnetic MFM probe gliding over the sample surfaces during AFM and MFM measurements caused peak shifts for the weak magnetic domains, which appeared as jitter in the high-frequency range of the PSD curves.
* The order of the second microstructure measurement process and the first magnetic domain measurement process, between the first microstructure and final magnetic domain measurement processes, should be swapped, and a gliding step should be added between them. This offsets the peak skewing of the magnetic domains caused by the weakly polarized probe during MFM measurement processes, without incurring excessive replacement costs.

## Figures and Tables

**Table 1:** The coercive forces of each sample.

Sample Index	A	B	C
Coercive force/(Oe)	0.786867	0.605464	0.552709

## Data Availability

The data is confidential.
